# Radiometric Identification of Signals by Matched Whitening Transform

**DOI:** 10.3390/s21248398

**Published:** 2021-12-16

**Authors:** Bijan G. Mobasseri, Amro Lulu

**Affiliations:** 1 Department of Electrical and Computer Engineering, Villanova University, Villanova, PA 19085, USA; 2 KMB Telematics Inc., Arlington, VA 22209, USA; amrlulu@gmail.com

**Keywords:** radiometric identification, RF fingerprinting, signal classification, whitening transform

## Abstract

Radiometric identification is the problem of attributing a signal to a specific source. In this work, a radiometric identification algorithm is developed using the whitening transformation. The approach stands out from the more established methods in that it works directly on the raw IQ data and hence is featureless. As such, the commonly used dimensionality reduction algorithms do not apply. The premise of the idea is that a data set is “most white” when projected on its own whitening matrix than on any other. In practice, transformed data are never strictly white since the training and the test data differ. The Förstner-Moonen measure that quantifies the similarity of covariance matrices is used to establish the degree of whiteness. The whitening transform that produces a data set with the minimum Förstner-Moonen distance to a white noise process is the source signal. The source is determined by the output of the mode function operated on the Majority Vote Classifier decisions. Using the Förstner-Moonen measure presents a different perspective compared to maximum likelihood and Euclidean distance metrics. The whitening transform is also contrasted with the more recent deep learning approaches that are still dependent on feature vectors with large dimensions and lengthy training phases. It is shown that the proposed method is simpler to implement, requires no features vectors, needs minimal training and because of its non-iterative structure is faster than existing approaches.

## 1. Introduction

Radiometric identification is the problem of attributing a signal to the source; often brand or model. Source identification is accomplished by RF fingerprinting of devices by looking for signatures that may arise from manufacturing tolerances, imperfections or normal statistical variations in production. There is considerable work in signal classification and modulation recognition [1,2]. However, radiometric identification does not neatly fit in either of the two categories. In many ways, radiometric identification is a more difficult problem as signals originating from different sources may have similar characteristics such as modulation, bit rates, pulse shapes, etc. This fact makes subtle device variations the main signature for radiometric identification. Such variations, however, are small, imperceptible and difficult to model. Why radiometric identification is of interest are many fold. The military has been interested in this capability for some time as a means of identifying friendly from hostile radar [3,4]. Satellite communication may be faced with intentional or unintentional jamming from rogue sources. Knowing the source and the brand of the interferer may help identify the offending source. Radiometric identification is also a valuable tool in securing wireless devices. Spoofing attempts in wireless networks and IoT devices can be thwarted if the source of the signal could be identified and blocked [5,6]. It is more difficult to mimic device characteristics that are embedded in signals than to replicate modulation or pulse shaping.

Radiometric identification can be formulated in the context of a statistical classifier. The classical approach follows feature extraction, dimensionality reduction by techniques such as PCA and finally multiple discriminant analysis classifier [7,8]. In [9], Square Integral Bispectra (SIB) is used to extract the unique stray features of individual transmitted signals, followed by PCA to extract a low-dimensional feature vector. It has been observed that features retained after dimensionality reduction are not necessarily optimal for classification.

A combined optimization of dimensionality reduction and fingerprint classification is proposed in [10]. The idea is to drive dimensionality reduction by minimizing the classification error and maximizing the mutual information between the reduced dimensionality features and the class label simultaneously. The RF fingerprint features are extracted from the statistics of the normalized instantaneous amplitude, phase and frequency of the signal resulting in feature vectors with up to 960 dimensions. The dimensionality reduction problem remains, however. Feature extraction for transmitter identification algorithms have been developed to operate in either transient [11] or steady state phases [12]. The transient phase is an analog state of the signal occurring right after the transmitter is activated whereas the steady state phase is characterized by modulation.

More recent work on radiometric identification has been influenced by the rise of deep learning (DL) tools. Examples are RF fingerprinting [13], IoT device fingerprinting [14], spectrum sensing [15] and RF device identification in cognitive networks [16]. What is still needed in all such work is the extraction of feature vectors followed by time consuming dimensionality reduction. The feature vectors extracted in [10], for example, have 960 dimensions before dimensionality reduction. In other words, the main problem still remains. The use of DL is often accomplished by the programming of off-the-shelf tools or use of various convolutional neural networks (CNN) routines implemented in Matlab. For example, the compressed bispectrum is identified as the feature then used to train a three layer CNN [17]. What differs are the number of layers, taps, filters, activation functions etc. Another example along this vein appears in [18] where Keras API is used with TensorFlow on the backend to distinguish distracted drivers. In [15], DL is implemented for RF device fingerprinting in the cognitive Zigbee networks using the time-domain complex baseband error signal as training and test data.The results show good accuracy (≈90%) but at high SNR (≥20 dB). In [19], the input data are preprocessed as Hilbert spectrum gray-scale images and achieves acceptable accuracy under moderate SNR levels (Avg 70% accuracy rate for SNR of 15 dB). A comprehensive performance comparison is shown for various DL algorithms in [13], reporting an average accuracy of 98% measured for 12 transmitters.

The fact that ML operates on much smaller data sets and requires much less training time compared to DL (hours of training [15]), provides more versatility to signal characteristics changes that occurs under different environmental circumstances (overheating, excess current, etc.), which can strongly affect the classification selected feature. This property of ML (data-driven) allows for fast feature update and consequently results in higher accurate classification on the long term. In addition, the reduced complexity compared to DL allows to easier hardware implementation and fast on-the-fly classification.

Specific Emitter Identification(SEI) is another paradigm for radiometric identification [20,21,22]. The SEI approach attempts to identify the unique transmitter of a signal using only external feature measurements [22]. SEI is implemented in two stages, (1) transient signal state and (2) steady state signal state. The transient approach applies to the particular signatures embedded in the signal as the transmitter powers up or down [23,24]. Transient approaches are more difficult to implement due to the unavailability or transient nature of the data that is often not accessible or saved. Steady state approach refers to the period where transients have stabilized. The available features include modulation and preamble [25,26], among others. In modulation-based techniques, the received and the target constellations are compared where the difference creates an RF fingerprint [27]. A fast decision identification algorithm appears in [28]. Identification is based on the similarity of a signal vector and its comparison to patterns available in a database. The approach is classified as an example of SEI applied to radar identification. The algorithm was applied hundreds of radar signals records which came from several different types of radars. In some cases, copies of the same type of radar of were investigated. Weighing all features equally, 85% correct recognition rate is reported for radar types. A mixed method of radar identification based on electromagnetic emission and intrapulse analysis appears in [29]. The premise is that electronic devices impart electrical features on the transmitted pulse. The signal model is *N* non-overlapping pushes form *K* transmitters. Linear Discriminant Analysis is used. Four distance metrics are used to classify the unknown pulse. It is reported that three copies of the same type of radar are successfully recognized.

Radiometric identification of communication protocols are also of interest. Identification of sources that use the LTE protocol is reported in [30,31]. The identification is based on unique modulation characteristics exhibited by the transmitters, resulting from minute imperfections introduced during radio hardware manufacturing. Device imperfections have been used as a signature for radiometric identification including clock jitter [32], the digital-to-analog converters (DAC) errors [33], local frequency synthesizer [34], the power amplifier non-linearity [35,36,37]. Power amplifier imperfections are also used for source identification [38]. Real radar signals are used for emitter identification [39].

An entirely different application for radiometric identification is radar. Even though the transmitters may belong to the same type of radar, they may exhibit subtle differences in their transmitted pulses. In [33], 18 features are used to identify three class of radars. Five radar emitter identification fingerprints based on radar signal transients are compared. Traditional techniques include radio frequency (RF), pulse amplitude, pulse width, intentional pulse modulation type, or pulse repetition intervals. In [40], unintentional modulation information on the emitter waveform is used as RF fingerprints, to tie the received signal and its corresponding the emitter. Unintentional Modulation on Pulse (UMoP) is a method that exploits variations due to manufacturing differences of the transmitter hardware, including the power amplifiers UMoP is like a fingerprint of an emitter and can identify transmitters from the same model [41]. Variational Mode Decomposition to radar identification is reported in [42]. The data set consist of 47 emitters. Some of these emitters were productions of the same radar. Results demonstrate that the effective SNR value should be around 47 dB to obtain a correct classification probability larger than 0.9.

In this work, the whitening transform is used as the framework for radiometric identification. This is fundamentally different than multiple discriminant analysis or deep learning. Identification is featureless, meaning it operates on raw complex IQ samples. Dimensionality reduction doesn’t apply as the IQ data are two dimensional to begin with. Therefore, the costly feature extraction and dimensionality reduction common to most radiometric identification techniques is avoided. As a whitening detector, radiometric identification differs from multiple discrimination analysis in one key metric. The metric is the degree of whiteness of the transformed data whereas the metric in multiple discriminant analysis is maximum likelihood driven by the distance metric. The distance measure, Förstner-Moonen distance plays a key role in establishing the whiteness of the whitened data. This metric is the input to a mode function followed by the Majority Vote Classifier.

## 2. Framework for Radiometric Identification

The received signal is first corrected for phase offset, oscillator frequency offset and symbol timing errors before application of the whitening transform. The whitening transformation is an orthogonal projection based on a variation of the PCA and is related to the orthogonal subspace projection [43]. One whitening transformation matrix per source is estimated from the training data. There is no need to know the modulation type, frequency, phase or anything else about the signal. Identification of the unknown source is based on the observation that a data set is “most white” when projected on its own whitening matrix than on any other, hence *matched whitening*. Projection of the unknown data on the whitening transforms whitens the data only if there is a match between the whitening matrix and the data. Even when the data matches its own whitening transform, the projected data is never truly white. A “whiteness” measure is developed by choosing a divergence metric for the comparison of covariance matrices. This measure is the sum of the squared logarithms of joint eigenvalues of the reference and test covariance matrices; the Förstner-Moonen distance. Whitening is well known in signal detection and it is often formulated as the Whitening Matched Filter. The goal is to decorrelate noise samples at the filter output. A 3D implementation of WMF is used for environmental impact studies in hyperspectral imagery [44]. Object detection by using whitening/dewhitening to transform target signatures in multitemporal hyperspectral appears in [45]. Examples of such whitening approaches mostly apply to signal and object detection and are not relevant to radiometric identification as proposed here.

### 2.1. The Whitening Transform

Let $X\in R^{p\times n}$ be the data matrix consisting of *n* measurements of *p* variables with the covariance matrix Σ. Statistical whitening is a linear transformation that transforms the data such that the covariance matrix of Y=WX is the identity matrix. The whitening transform matrix is not unique. In fact, [46] mentions five different projection matrices that whiten the data, with the most prominent ones being the PCA and ZCA whitening [47]. Specifically,

(1) $$\begin{array}{cc} W^{PCA} & =\Lambda^{-\frac{1}{2}}U^{T} \end{array}$$

where U and Λ are the matrices of eigenvectors and eigenvalues in the decomposition of the covariance matrix $\Sigma ={U\Lambda U}^{T}$. The whitening transformations produce decorrelated data but to what end? More importantly, what role does whitening play in radiometric identification? This is where the matched whitening transform deviates from the existing use of PCA in radiometric identifications. PCA is best known for data compression by guiding the removal of the components of Y with insignificant energy. The features that remain are not necessarily the best for classification. Yet, almost all PCA-based radiometric classification techniques use the features that survive compression in a subsequent discriminant function to classify the data. ZCA has the added property of zero-phase by undoing the rotation caused by the PCA. Neither of the two are applicable here. Producing uncorrelated data is a preprocessing step from which lower dimensionality feature vectors are extracted. Dimensionality reduction does not apply to IQ samples as there are only two dimensions to begin with and are largely decorrelated already. PCA has been used in deep learning as well by accelerating the convergence in convolutional neural networks [48].

### 2.2. Classification by Matched Whitening

The data are organized in an N×M matrix $X=\left[x_{1},x_{2},\ldots ,x_{M}\right],x_{i}\in R^{N\times 1}$ where *M* is the number of measurements and *N* is the number of variables, or dimensions. For the IQ data, N=2 and *M* is the number of symbols in the record. Let $W_{i}$, i=1,2,…,m be the whitening transform matrices for *m* source signals $\left\{c_{1},c_{2},\ldots ,c_{m}\right\}$. The class-dependent whitening matrices are computed offline from the training data. Since the IQ data are affected by phase and frequency offsets, the data need to be corrected before the whitening matrices are calculated. The test data are partitioned into blocks used to generate statistics. There is no “correct” block length. It depends on the rate of change of phase, frequency offset or Doppler shift. In the case of nonlinear phase offset, block lengths are chosen short enough to insure near stationary phase during phase estimation. More on how to choose the block length for reversing the frequency offset appears in Section 3.

Let $X_{j}\in R^{2\times M}$ be the *j*th block. The unknown measurement vector is repeatedly whitened by $W_{i},\forall i$.

(2) $$\begin{array}{c} \begin{array}{cc} Y_{i} & =W_{i}X_{j},i=1,2,\ldots ,m\\ cov(Y_{i}) & =I\Leftrightarrow X_{j}\in c_{i} \end{array} \end{array}$$

The covariance matrix of the whitened data is an identity matrix if and only if $W_{i}$ matches the data that are projected on it. In other words, the whitening matrix can only whiten its own data. Conversely, if the unknown data are whitened, the data belongs to the same class that the whitening matrix came from.

**Figure 1:**
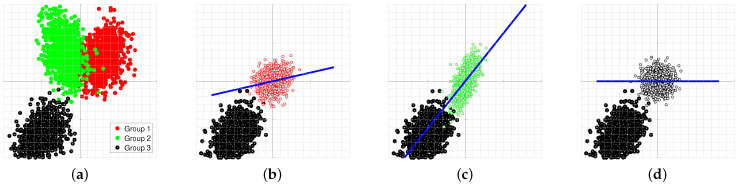
The “unknown” data, shown in black, are repeatedly projected on the spaces spanned by the eigenvectors of other groups. When the principal axis of the projected data aligns with the reference axis, a match is declared. In this example, the source of the unknown data is the group shown in black. (**a**) Three multivariate normal data sets simulating different signal sources, (**b**) Black data is whitened by the red whitening matrix, (**c**) Black data is whitened by the green whitening matrix, (**d**) Black data is whitened by its own whitening matrix.

**Figure 2:**
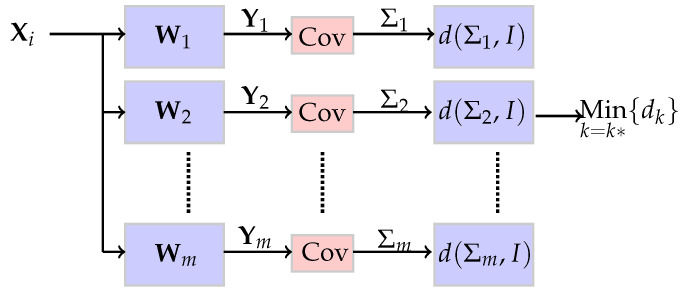
Radiometric identification by whitening transformations. The branch with the most whitened data reveals the source of the unknown signal.

To illustrate this point, three multivariate normal populations are created and shown in Figure 1a. The 3rd data set (in black) is used as the “unknown” source and is repeatedly projected on $W_{i},i=1,2,3$. After each projection, the scatter diagram is plotted and shown in Figure 1b–d. When the data from group 3 is whitened by $W_{1}$, Figure 1b, the major axis of the projected data appears at an angle to the principal axis of the projection matrix. This indicates that the data and the whitening matrix are mismatched. Repeated projections produce Figure 1b–d. It is only in Figure 1d that the whitening transformation produces a circular scatter diagram. The projection that produces the least correlated data identifies the brand. This property indicates that the source of the unknown data matches the whitening transform of group 3. The detector can be implemented as a bank of parallel matched filters shown in Figure 2.

### 2.3. Development of a Whitening Measure

There are several issues with tying the unknown data to its own whitening matrix. First, the IQ components of the real data are already quite decorrelated so whitening may not bring significant additional decorrelation. Second, the subspace defied in (1) is created offline from the training data. However, the test data are different even if coming from the same population as the training data. If the data different than the training set are used, the whitening of the data will be approximate. The core property is that the covariance matrix of the unknown data will resemble the identity matrix if projected on its own subspace more than on any other. Third, how to measure “whiteness”. This is a problem in covariance matrix matching [49].

**Figure 3:**
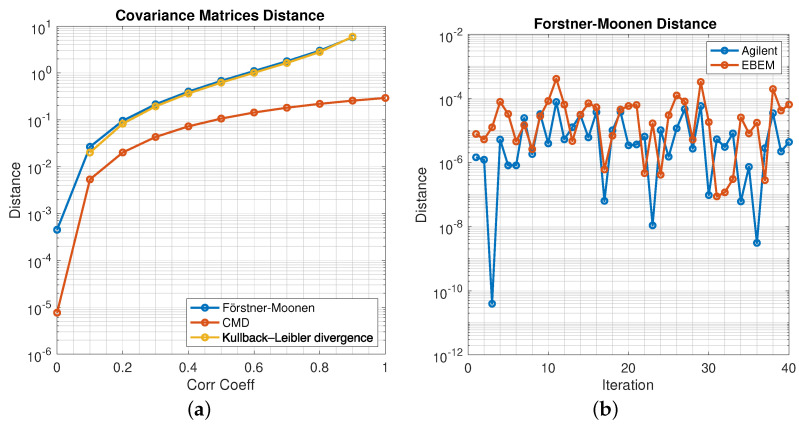
Two illustrations of the effectiveness of the Förstner-Moonen distance. All three similarity measures behave monotonically with correlation with the Kullback-Leibler measure virtually coincident with Förstner-Moonen. (**a**) Comparison of Förstner-Moonen, CMD and Kullback-Leibler distance metrics, (**b**) The Förstner-Moonen distance is lower when data are transformed by matched whitening transfom.

There are any number of metrics to measure the distances between two symmetric, positive definite covariance matrices. They include KL divergence, Euclidean distance, squared Frobenius norm, Bhattacharyya distance, Bregman matrix divergence and LogDet [50], among others. In this work we use the Förstner-Moonen metric [49] as a similarity measure of two covariance matrices. As a point of reference, the well-cited Correlation Matrix Distance(CMD) metric [51] and the Kullback-Leibler measures are studied. There is no one definition for similarity but three are monotonic with correlation and hence are valid measures. We have superimposed CMD, KL and Förstner-Moonen plots for comparison. The graphs appear later on in Figure 3a. As expected, the pairwise distance increases with increasing correlation, meaning that the covariance matrix of correlated variables is at farther distances from a diagonal covariance matrix. It is noteworthy that the KL measure is virtually coincident with the Förstner-Moonen metric hence justifying its use as a similarity index.

Let A and B be the reference and measured covariance matrices. The proposed distance measure is defined by,

(3) $$\begin{array}{c} d\left(A,B\right)=\sqrt{\sum_{i=1}^{n}ln^{2}\lambda_{i}\left(A,B\right)} \end{array}$$

where $\lambda_{i}\left(A,B\right)$, the joint eigenvalues of A and B, are the roots of |λA−B|=0. In the context of the whitening transform, the reference covariance matrix is the identify matrix A=I and $B=cov(Y_{i})$ is the covariance matrix of the unknown data whitened by $W_{i}$. Therefore, the joint eigenvalues reduce to simply the eigenvalues of the measured covariance matrix B of the unknown data.

The classifier built on (3) is a Majority, or Plurality, Vote Classifier [52] governed by rules $h_{1},h_{2},\ldots ,h_{m}$. The rules are membership functions. Given the measurements $X_{i}$ from an unknown source,

(4) $$\begin{array}{c} \begin{array}{cc} h_{j}\left(X_{i}\right) & =1\;X_{i}\in c_{j}\\ \; & =0\;X_{i}\notin c_{j} \end{array} \end{array}$$

The membership functions work off the Förstner-Moonen distance as follows,

(5) $$h_{j}\left(X_{i}\right)=\left\{\begin{array}{cc} 1 & d\left(\Sigma_{j},I\right)<d\left(\Sigma_{k},I\right)\;\forall k\neq j\\ 0 & \mathrm{otherwise} \end{array}\right.$$

Every time a whitened block is closer to its own true class as measured by the Förstner-Moonen distance, a 1 is recorded. The rule outputs are then fused in the following way,

(6) $$\begin{array}{cc} C(X_{i}) & =mode\left\{\sum_{i=1}^{p}h_{1}\left(X_{i}\right),\sum_{i=1}^{p}h_{2}\left(X_{i}\right),\ldots ,\sum_{i=1}^{p}h_{m}\left(X_{i}\right)\right\} \end{array}$$

where *p* is the number of blocks. The mode function is the number that occurs most often in the set, i.e., $h_{j}\left(X_{i}\right)$ is the number of times $X_{i}$ is voted to belong to $c_{j}$. The unknown measurement $X_{i}$ is classified to the class receiving the most votes. This process is pictured in Figure 2. This is an example of “hard” voting. The alternative is “soft” voting where the frequency of assignments to classes are retained.

The computational complexity of the algorithm consists of the whitening matrix, whitening transform and eigenvalue decomposition. If $X\in R^{d\times M}$, where *d* is the number of variables and *M* is the number of measurements, the complexities of the whitening transform is $O(d^{2}M+d^{3})$, whitening transform is $O(d^{2}M)$ and eigendecomposition is $O(d^{3})$. With IQ signal representation, d=2 and it’s constant throughout. Therefore, each of the complexities above ultimately reducing the overall complexity to O(M). i.e., linear with the number of measurements.

## 3. Reversing Phase and Frequency Offsets

**Figure 4:**
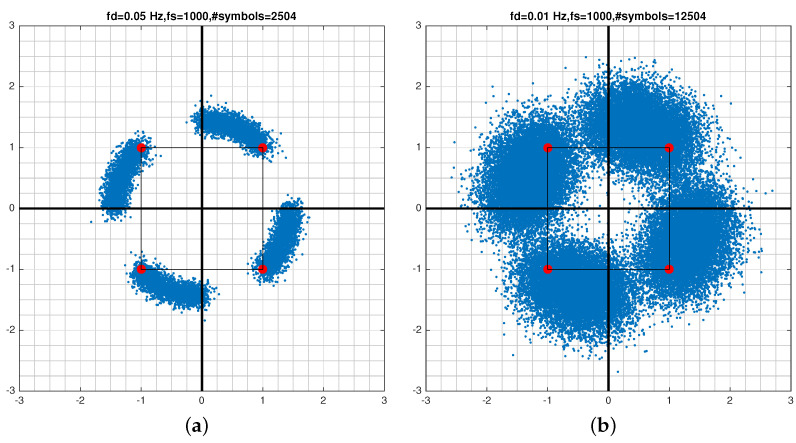
Constellation smearing caused by time-varying phase offset under two SNR values. (**a**) SNR = 20 dB, (**b**) SNR = 10 dB.

The first challenge to radiometric identification surfaces before the algorithm is implemented. Signals are often made available with uncorrected phase rotations. There are two types of rotations. Fixed rotation is cause by a constant phase offset of the reference carrier. Time-varying rotation is caused by the frequency mismatch of the reference carrier. The mismatch could be hardware-related or caused by Doppler. Either way, it is an unknown quantity. The frequency mismatch, termed *offset frequency* $f_{d}$, causes a corresponding time-varying phase which results in constellation smearing. This is different than fixed phase offset which causes the entire constellation to rotate. Figure 4 shows the time varying phase offset under two SNR levels. Both fixed and time varying rotations must be reversed prior to radiometric identification.

### 3.1. Background

Phase and frequency offset correction prior to source identification is not always addressed in the radiometric identification literature [17]. The traditional approach to carrier phase recovery is the power law method [53]. Raising the signal to the *M*th power creates a tone at *M* times the offset frequency which can be used to derotate the constellation. However, this method only works for fixed phase offsets. The approach presented here extracts arbitrary phase trajectories by fitting a model to the maximum likelihood estimate of phase points measured over multiple signal segments. The phase trajectory is first estimated from signal segments that are short enough for the phase to be considered stationary; essentially a snapshot of the phase in time. The slope of the line fitted to the phase angles using least squares is proportional to the offset frequency. In addition, the least squares fit method handles nonlinear phase trajectories caused by second order offset frequency effect. This is not possible with he power law method.

### 3.2. Signal Model

An MPSK signal is modeled as follows

(7) $$\begin{array}{cc} s(t) & =\sqrt{P}e^{j(2\pi f_{c}t+\phi_{m})}+n\left(t\right),m=0,1,\ldots ,M-1 \end{array}$$

(8) $$\begin{array}{cc} \phi_{m} & \in \{\frac{2\pi m}{M},m=0,1,\ldots ,M-1\} \end{array}$$

where *P* is the received carrier power, $f_{c}$ is the carrier frequency and $\phi_{m}$ is the original constellation vertices. The local oscillator offset frequency $f_{d}$ creates a time varying phase offset $\theta \left(t\right)=2\pi f_{d}t$. Therefore, the offset frequency is the the slope of the phase trajectory. The basebanded signal for the *k*th symbol is

(9) $$\begin{array}{c} r_{k}=i_{k}+jq_{k}=\sqrt{P}e^{j(\phi_{k}+\theta_{k})}+noise \end{array}$$

The discrete model for the phase offset is $\left\{\theta_{k}=2\pi f_{d}t,t=kT_{s},k=1,2,\ldots \;K\right\}$ where $T_{s}$ is the symbol length and *K* is the number of symbols in the block used to estimate phase rotation. Successive symbols rotate by $2\pi f_{d}T_{s}$ radians away from their nominal positions. This movement forms an arc over time thus causing a smearing effect shown in Figure 4. To correct for this rotation, an estimate of $\theta_{k}$, ${\hat{\theta }}_{k}$, must be found and used to recover $f_{d}$ and derotate the block of symbols. The maximum symbol rotation over a block is $T=KT_{s}$.

Offset frequency estimation can be achieved by first estimating the phase trajectory. The estimation of θ(t) is performed over short blocks of length *T* to assure phase stationarity, i.e., $\left\{\theta \left(t\right)\approx \theta_{k},t\in T\right\}$. Therefore, there is one phase estimate per block of data. The quantity $f_{d}T$ is the fractional rotation of the constellation over 2π for the block length *T*. This quantity must be kept small for two reasons. One, smaller $f_{d}T$ means a finer sampling of the phase curve. This is important in capturing phase nonlinearity by piece-wise linear modeling. Two, large $f_{d}T$ pushes the symbols beyond their original symbol quadrant. This effect can be seen in Figure 4b where symbols in the first quadrant have been pushed to the second quadrant. What constitutes short or long segments is explained in the following section.

## 4. Results

This section implements the proposed radiometric identification using simulated and real data. First, data are corrected for offset frequency and used to reverse the time varying phase offset. Second, the proposed algorithm that is governed by rule (6) is implemented producing confusion matrices.

### 4.1. Signal Phase and Offset Frequency Correction

Data are simulated for a QPSK signal subject to local oscillator frequency offset. Table 1 shows the simulation parameters.

The phase curve is built from the estimates of the instantaneous phases computed from signal blocks short enough to insure stationary phase. Each block of data generates one phase estimate. Multiple blocks define a segment where symbols rotate a maximum of 5.62°.

**Figure 5:**
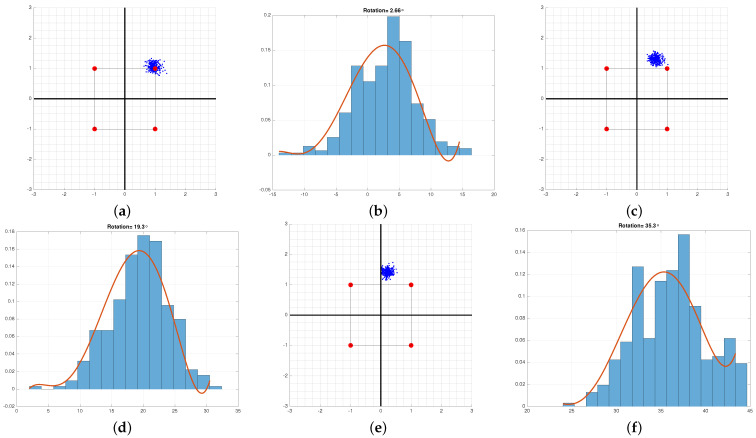
Phase trajectory build by estimation of phase rotation per block. Block lengths are chosen short enough to keep the phase trajectory linear. (**a**) Phase offset snapshot 1, (**b**) Phase offset historgram used for phase estimation, (**c**) Phase offset snapshot 2, (**d**) Phase offset historgram used for phase estimation, (**e**) Phase offset snapshot 3, (**f**) Phase offset historgram used for phase estimation.

**Figure 6:**
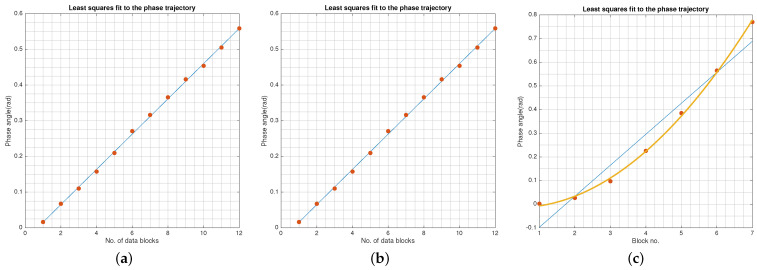
The offset frequency $f_{d}$ is the slope of the phase trajectory $\theta =2\pi f_{d}t$. The least squares fit to the estimated phase values is used to estimate the slope. ${\hat{f}}_{d}=0.0505$ with $f_{d}=0.05$. This approach works for nonlinear phase trajectory as well. (**a**) SNR = 20 dB, (**b**) SNR = 10 dB, (**c**) Quadratic fit to time varying phase angle.

Figure 5 shows the process by which instantaneous phase values are gathered and used in the model fitting step. This step can also be explained as the sampling of the phase curve. The symbol phases per block are histogrammed followed by fitting a polynomial. The peak of the polynomial is ${\hat{\theta }}_{k}$ for the *k*th block. This step is repeated over multiple blocks and shown in Figure 5a–f. The estimated phases $\left\{{\hat{\theta }}_{k},k=1,2,\ldots ,M\right\}$ define the linear phase trajectory the slope of which determines $f_{d}$. Figure 6 is the least squares fit of the phase model to the data. Figure 6a,b correspond to SNR = 20 dB and 10 dB, respectively. Figure 6c illustrates that a nonlinear phase trajectory can be modeled and tracked as well. The estimated ${\hat{f}}_{d}=0.0505\;\mathrm{Hz}$ and ${\hat{f}}_{d}=0.0455\;\mathrm{Hz}$ at SNR = 20 dB and 10 dB, respectively. The true offset frequency is 0.05 Hz.

**Figure 7:**
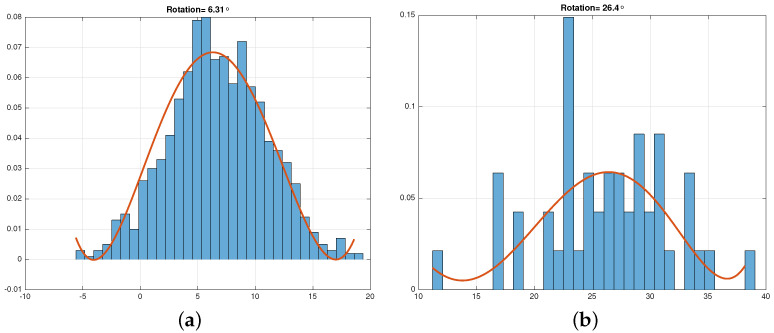
Phase offset histogram is unimodal for small $f_{d}T$, (**a**). Longer data blocks cause phase smearing illustrated by a broad phase angle histogram, (**b**). (**a**) Small phase offset, 6°, (**b**) Large phase offset, 26°.

Symbols rotate by $2\pi f_{d}T$ radians over the length of a block. This rotation must be kept to a small fraction of the quadrant that the symbols belong to. For example, in QPSK, each quadrant is π/2 radians. The proper block length is guided by the modality of phase histograms. A unimodal phase histogram with a distinct peak indicates that phase variations remain close to the nominal value, Figure 7a. For large $2\pi f_{d}T$, either due to large $f_{d}$ or long block length *T*, the histogram becomes multimodal with no distinct peaks, Figure 7b. Another disadvantage of large $f_{d}T$ is the 2π phase ambiguity where symbols move around the circle multiple periods.

### 4.2. Radiometric Identification

**Figure 8:**
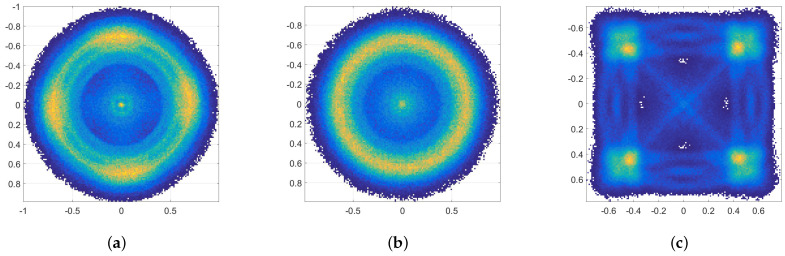
The signal constellation for EBEM signals suffers smearing due to varying amount of $f_{d}T$, (**a**,**b**). Smearing is completely reversed after phase offset correction. (**a**) Limited phase offset, (**b**) Phase offset of multiples of 2π, (**c**) Constellation after phase and frequency offset correction.

**Figure 9:**
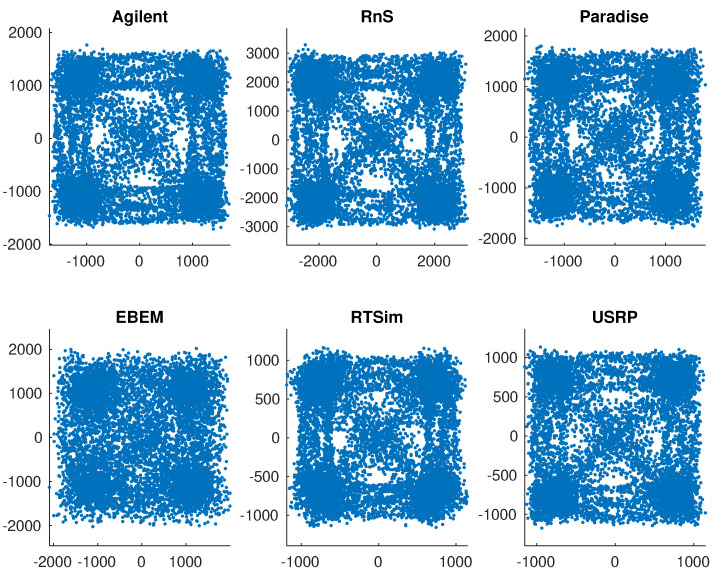
Six constellations from six different sources are corrected for phase and frequency offsets and used for training and testing. There is noise of unknown strength already in the data. Constellation similarities illustrate the difficulty of radiometric identification by feature extraction.

We now apply the proposed radiometric identification method to the signals generated by the following waveform generators or standards: Agilent [54], Viasat EBEM [55], Teledyne Paradise [56], KRATOS Real Time Channel Simulator (RTsim) [57], and USRP [58]. The data have QPSK modulation sampled at 2.95 MHz for a total of 35 million symbols per model. Figure 8a,b show signal constellations that are affected by varying amount of smearing. Figure 8b is a particularly sever case due to the large $f_{d}T$ product causing symbols to rotate potentially multiples of 2π. Following the estimation of $f_{d}T$ and derotation of symbols, the original constellation is restored in Figure 8c. Figure 9 is a close up of six constellations after all phase and frequency offsets have been removed. The task now is to attribute the signals to individual sources. Given the similarity of the constellations in structure and features, it is clear that radiometric identification is a much more challenging problem than conventional signal classification based on modulation information.

### 4.3. Class Confusion Matrices

Training the classifier involves the computation of 5 matched whitening matrices, $W_{i},i=1,2,\ldots ,5$. The data consists of 35 million symbols taken from QPSK modulated signals originating from five different radios. The training set consists of $5\times 10^{5}$ symbols which is about 1.4% of the total data. The Majority Vote Classifier needs a voting scheme. Votes are generated by dividing the data into 72 blocks of $5\times 10^{5}$ samples each. Each block generates one vote which is then tabulated over the entire signal length. The test blocks are drawn from an “unknown" source, corrupted by Gaussian noise and repeatedly projected on whitening matrices corresponding to each source. The Förstner-Moonen distance is used to compute the mode function in (6) leading to the compilation of the confusion matrices.

Before creating the confusion matrices, the behavior of the Förstner-Moonen distance measure needs to be studied. According to (3), as the process is increasingly whitened, the Förstner-Moonen distance between the whitened covariance matrix and the identity matrix is narrowed. The theoretical minimum distance is zero for white noise. To test for this behavior, two random variables with adjustable correlation coefficients are created and placed in a two-column matrix. The covariance of this matrix is calculated as a function of correlation values and the corresponding Förstner-Moonen distance is plotted. The results are plotted in Figure 3. As Figure 3a shows, the distance is an increasing function of correlation, reflecting that the covariance matrix is moving away from that of a white noise process for increasing correlation. This is expected.The second property of the Förstner-Moonen measure is that the unknown data is closer to a white noise process when whitened by its own whitening transformation than any other, hence matched whitening. To show this property, the data from Agilent is whitened by its own whitening matrix and then by the whitening matrix of Viasat EBEM. Distance calculations are performed over 40 blocks of data and plotted in Figure 3b. What stands out is that the Förstner-Moonen distance for the Agilent data is almost always less than that when the Viasat EBEM whitening matrix is used. This behavior is expected, meaning that a correct decision is made every time it happens. This count is essentially the basis for populating the confusion matrices over all sources.

Following the above observations, the corresponding confusion matrices can now be computed and are shown in Table 2. The numbers indicate the percent of correct votes cast for each source over 72 frames of the test data. Note that the mode classifier in (6) looks for a plurality of the votes to pick a winner. It’s a hard voting scheme. For example, Paradise has received only 77.1% of the votes but the unknown signal is still correctly classified to Paradise. Therefore, Table 2 indicates 100% correct classification. Confusion matrices can be used in a soft voting scheme as well by keeping the actual vote percentages.

**Figure 10:**
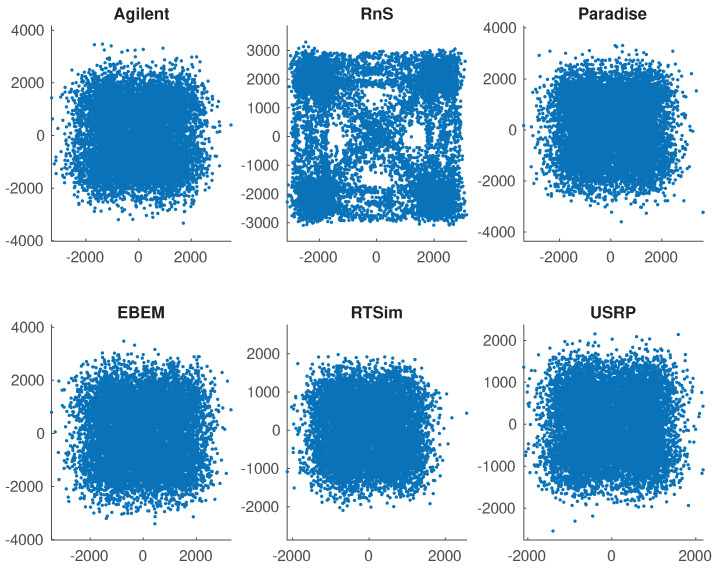
Constellations in SNR = 5 dB noise. Constellation structures have practically disappeared, yet the source identification is still largely possible.

Next, we investigate the impact of smaller data sets and added noise above and beyond what is already in the data. The total sample size is now $10^{7}$ which are broken into blocks of a quarter million samples each translating to less than 100 msec. This length generates 40 blocks that are used to get classification statistics in the form of confusion matrices. Table 3 shows the results @ SNR = 15 dB added Gaussian noise. This is above and beyond what is already in the data. All sources are identified correctly except for KRATOS RTSim which is identified as Teledyne Paradise. Even then, the 2.5% difference is well within the statistical variations of the run. The precent correct classification numbers for each source show a large drop compared to Table 2 but the majority voting scheme still makes the correct decision, albeit at a reduced margin. For example, Agilent data are correctly associated with Agilent only 30% of the time but that is still higher than any others. Table 4 and Table 5 repeat the process for SNR = 5 dB and 0 dB. Even though the rates and margins are lower, the majority vote scheme still picks the correct class. When margins are low, statistical variability plays a role in making correct source identification. Notice that the large margin of USRP in Table 2 helps it largely maintain correct identification even at 5 dB SNR in Table 4. To show how dire the situation is, Figure 10 shows the constellation in SNR = 5 dB noise. The lack of identifying features is evident throughout. Note that RTSim and Paradise are tied. This difficulty is of course reflected in Table 4 as well but correct identification is still possible. Four out of five sources are correctly identified and the fifth one is tied. Table 5 is the extreme case of SNR = 0 dB. EBEM and Paradise are still correctly identified.

### 4.4. Comparisons

A comprehensive comparison of SVM, CNN and D(eep)NN are reported for six radios in [13]. The correct classification rates are 44.8% (SVM), 82.4% (CNN) and 71.9% (DNN). However, in the absence of accepted benchmarks for radiometric identification, which do not exist, pure numerical comparison are not conclusive. Factors such as the complexity of the algorithm, processing speed, training data size and other assumptions are considered, the comparison is difficult. Even the choice of radios or protocols are not common. The reported training sample size in [13] is 10% whereas it is 1.4% here. More importantly, no carrier recovery step reported. By assuming perfect phase and frequency alignment at the local oscillator, no mitigation for constellation smearing of the kind reported here has been carried out. This is a significant omission. There is also no noise in the system. Dealing with high dimensionality is another factor. The whitening transformation is featureless thus bypassing the dimensionality reduction whereas feature vectors extracted in [10] have 960 dimensions. RF device fingerprinting in the cognitive Zigbee networks shows good accuracy (≈90%) but at high SNR (≥20 dB) [15]. In [19], the input data are preprocessed as Hilbert spectrum gray-scale images, and achieves acceptable accuracy under moderate SNR levels (Avg 70% accuracy rate for SNR of 15 dB).

## 5. Conclusions

The problem addressed in this paper is the attribution of a signal to an unknown source. Previous approaches have been based on feature extraction, dimensionality reduction and some implementation of a minimum distance classifier. The approach here proposes the degree of whiteness of the transformed data as a signature for radiometric identification of the signal. It’s a featureless approach that skips feature extraction by using the raw IQ data. This formulation demands minimal computational load compared to PCA or deep learning methods. There are two other features that make the algorithm stand out. One is using real data captured by satellite radios. The other is addressing carrier and phase recovery by reversing the embedded phase and frequency offsets as a preprocessing step. Algorithms that are tuned to the data assuming perfect carrier capture will fail in practice. This work can be extended in a number of ways, such as expanding the radio source database to military and commercial radar, wireless broadcasts, modeling time varying frequency offsets and a more broad comparison with the competing deep learning methods.

## Figures and Tables

**Table 1:** Simulated data used for carrier recovery.

Item	Value
Modulation	QPSK
Symbol rate	1000/s
Block length	0.312 s
Symbols/block	3120
No. of blocks	8
Segment length	2.5 s
Total no. of symbols	2504
Max. rotation per block	5.62°
Total rotation per segment	45°
Offset frequency ($f_{d}$)	0.05 Hz
SNR	20 dB

**Table 2:** Five source cross classification rates.

	Agilent	Viasat EBEM	Paradise	RTSim	USRP
Agilent	**91.4**	0	0	8.6	0
EBEM	0	**100**	0	0	0
Teledyne Paradise	0	0	**77.1**	0	22.9
KRATOS RTSim	0	0	0	**100**	0
USRP	0	0	0	0	**100**

**Table 3:** Reduced data set Table 2 @ SNR = 15 dB.

	Agilent	EBEM	Paradise	RTSim	USRP
Agilent	**30.0**	22.5	12.5	0.0	22.5
Viasat EBEM	15.0	**85.0**	0.0	0.0	0.0
Teledyne Paradise	5.0	2.5	**77.5**	2.5	12.5
KRATOS RTSim	2.5	12.5	**22.5**	20.0	15.0
USRP	22.5	0.0	20.0	0.0	**57.5**

**Table 4:** Same as Table 3 @ SNR = 5 dB.

	Agilent	EBEM	Paradise	RTSim	USRP
Agilent	**22.5**	20.0	7.5	15.0	17.5
Viasat EBEM	10.0	**80.0**	2.5	0	5.0
Teledyne Paradise	15.0	0	**52.5**	12.5	10.0
KRATOS RTsim	12.5	20.0	**12.5**	**12.5**	15.0
USRP	35.0	2.5	20.0	2.5	**37.5**

**Table 5:** Same as Table 3 @ SNR = 0 dB.

	Agilent	EBEM	Paradise	RTSim	USRP
Agilent	17.5	**27.5**	17.5	5.0	10.0
Viasat EBEM	20.0	**60.0**	5.0	2.5	2.5
Teledyne Paradise	15.0	5.0	**27.5**	17.5	15.0
KRATOS RTsim	7.5	**22.5**	10.0	15.0	12.5
USRP	**20.0**	12.5	17.5	2.5	17.5

## Data Availability

Not applicable.
